# Characterization of Active MMP9 in Chronic Inflammatory Diseases Using a Novel Anti-MMP9 Antibody

**DOI:** 10.3390/antib12010009

**Published:** 2023-01-18

**Authors:** Maile Velasquez, Chris O’Sullivan, Robert Brockett, Amanda Mikels-Vigdal, Igor Mikaelian, Victoria Smith, Andrew E. Greenstein

**Affiliations:** 1 Gilead Sciences, Inc., Foster City, CA 94404, USA; 2 Corcept Therapeutics, 149 Commonwealth Dr, Menlo Park, CA 94025, USA

**Keywords:** active MMP9, MMP9 function, andecaliximab

## Abstract

Matrix metalloproteinase 9 (MMP9), a protease implicated in multiple diseases, is secreted as an inactive zymogen and requires proteolytic removal of the pro-domain for activation. The relative levels and functionality of the pro- and active-MMP9 isoforms in tissues are not characterized. We generated a specific antibody that distinguishes an active form of MMP9, F107-MMP9, from the inactive pro-MMP9 isoform. Using multiple in vitro assays and specimen types, we show that F107-MMP9 expression is localized and disease-specific compared with its more abundant parental pro-form. It is detected around sites of active tissue remodeling, including fistulae of inflammatory bowel and dermal fissures in hidradenitis suppurativa, and is expressed by myeloid cells, including macrophages and neutrophils. Together, our findings provide insights into the distribution and potential role of MMP9 in inflammatory diseases.

## 1. Introduction

Matrix metalloproteinase 9 (MMP9) has multiple described roles in key pathological processes, including angiogenesis, basement membrane degradation, and cytokine activation [1,2,3]. Diagnostic, disease-staging, and prognostic associations of MMP9 levels in various biospecimens have been reported [4,5,6,7,8,9,10,11,12]. Despite this trove of data on MMP9, study of the active form of the protease in human disease has been limited, likely due to a lack of assays and reagents that can reliably measure the active form of MMP9 and/or its half-life and rate of turnover or clearance. 

Like many proteases, MMP9 is secreted as an inactive zymogen and requires proteolytic removal of the pro-domain before catalysis [13,14]. The relative levels and functionality of the pro- and active-MMP9 isoforms in different tissues remain to be fully characterized. Zymography is sensitive, but it is ultimately impossible to assign a specific identity to each band in complex biological samples [15,16,17]. Chemical substrate-mimetic probes and inhibitors lack the ability to distinguish between MMP9 and other, closely related MMPs, such as MMP2 [18,19]. Immunoprecipitation coupled with labeled-peptide proteolysis assays is sensitive to endogenous MMP inhibitors, such as tissue inhibitor of metalloproteinases (TIMP) 1 or α2-macroglobulin, and detection of the active form of MMP9 when these inhibitors are present remains challenging [20].

The MMP9 expressed in most cultured cell lines is secreted almost exclusively in the inactive pro-form, and engineered destabilizing mutations are needed to generate appreciable levels of the active form in vitro [21]. Likewise, proteolysis of MMP9 in vitro is often assumed to be activating if it results in a proteolyzed product of the correct molecular weight; however, MMP9 can be proteolyzed without the precise pro-domain removal and thus remain inactive [22]. Widespread misunderstandings about the active form of MMP9 have contributed to contradictory conclusions in the literature and reproducibility challenges, confounding results about the function of this protease [15,23,24,25].

Therapeutic targeting of MMP9 in the clinical setting would be greatly assisted by highly sensitive assays to aid pharmacodynamics, indication selection, and/or patient stratification. A drug targeting a specific protease therefore could benefit from assays that can distinguish between the pro and active forms [26]. Andecaliximab (ADX, GS-5745) is a monoclonal antibody (mAb) that specifically inhibits MMP9 [27,28]. Early-phase clinical studies show that ADX, unlike broad-spectrum, small-molecule MMP9 inhibitors, is well tolerated in multiple inflammatory and oncology indications. Studies of this selective inhibitor in mice, humans, and in vitro systems have provided insights into the role of MMP9 [27,28,29,30].

In this report, we describe the generation of mAbs that measure an active form of MMP9, F107-MMP9, that can be used in multiple assays (Western blot, enzyme-linked immunosorbent assay [ELISA], and immunohistochemistry [IHC]) and multiple specimen types (tissue homogenate, serum, and formalin-fixed/paraffin-embedded [FFPE] tissues). Using these reagents and assays, we specifically characterized the F107-MMP9 species in active sites of chronic inflammatory diseases including fistulae of inflammatory bowel disease (IBD) and superficial fissures in the skin of hidradenitis suppurativa (HS). We identify distinctive expression patterns for F107-MMP9, including accumulation in areas of tissue remodeling by myeloid cells.

## 2. Materials and Methods

### 2.1. Antibody Generation and Characterization

Rabbits were immunized with the peptide (NH_2_)-FQTFEGDC conjugated to both ovalbumin and keyhole limpet hemocyanin. Based on serum titers toward the immunizing peptide, animals were selected for hybridoma library generation (Abcam, Burlingame, CA, USA). In total, 40,000 individual hybridomas were screened for immunoreactivity of supernatants with the immunizing peptide. Positive supernatants were screened by Western blot with full-length pro- and active-MMP9 recombinant standards [28]. Positive hybridoma supernatants were screened by IHC against a series of formalin-fixed HEK293 cell pellets containing active MMP9 (see Appendix A for details). Immunoglobulin G complementary DNA (cDNA) was sequenced from the positive hybridomas by 5′RACE (Abcam), cloned into pcDNA3.1 expression vector (Invitrogen, Thermo Fisher Scientific, Carlsbad, CA, USA), expressed in HEK293 cells, and purified by protein-A chromatography (GE Healthcare Life Sciences, Little Chalfont, Buckinghamshire, UK). Sandwich immunoassays were conducted by coating the plate in 1 μg/mL recombinant antibody, adding recombinant MMP9 standard ADX, detecting with total MMP9 antibody (MAB936; R&D Systems, Minneapolis, MN, USA), and quantifying with a ruthenium-conjugated anti-mouse antibody (Meso Scale Discovery, Rockville, MD, USA).

### 2.2. In Vitro MMP9 Activation

Recombinant active MMP7, MMP12, pepsin A, or plasmin (R&D Systems) was titrated against recombinant pro-MMP9 in MMP9 reaction buffer (150 mM NaCl, 10 mM Hepes pH 7.5, 0.05% brij-035, and 5 mM CaCl_2_). Reactions were analyzed by Western blots simultaneously probed with anti-total MMP9 (catalog [Cat] #819701; BioLegend, San Diego, CA, USA) and anti-F107-MMP9, and visualized with fluorescently labeled anti-mouse (LI-COR Biosciences, Lincoln, NE, USA) and anti-rabbit (LI-COR Biosciences) secondary antibodies. The SeeBlue Plus2 molecular weight marker was used for all Western blots (LC5925, Thermo Fisher Scientific, Carlsbad, CA, USA).

### 2.3. Assessment of Active MMP9 in Human Tissues

HS specimens were collected by Trans-Hit Bio (Quebec, Canada). Matched frozen and FFPE tissues were collected after whole colectomy (Folio BioSciences, Powell, OH, USA) from patients with ulcerative colitis or Crohn’s disease. As a control, non-diseased tissue adjacent to sites of active disease was also collected. Frozen tissues were lysed using an Omni Bead Ruptor 24 (Omni International, Kennesaw, GA, USA; Cat #19-040) and subjected to Western blot as described above. 

FFPE samples were sectioned at 5 µm and mounted on positively charged glass slides. Slides were immunolabeled using the Ventana DISCOVERY ULTRA autostainer (Ventana Medical Systems, Tucson, AZ, USA) with the following procedure: 

(Reaction buffer washes [Ventana Medical Systems; Cat #5353955001] between each step)

1. Deparaffinization with EZ Prep (Ventana Medical Systems; Cat #5279755001) for 12 min at 69 °C;
2. Antigen retrieval with CC1 cell conditioning (Ventana Medical Systems; Cat #6414575001) for 64 min at 95 °C;
3. ChromoMap Inhibitor (Ventana Medical Systems; Cat #5266645001) for 8 min at room temperature;
4. Manual addition of 100 µL of AB006988 at a concentration of 5 µg/mL for 1 h at room temperature;
5. Manual addition of 100 µL Mach 3 Anti-Rabbit Probe (Biocare Medical, Concord, CA, USA; Cat #RP531) for 30 min at room temperature;
6. Anti-Mouse HQ (Ventana Medical Systems; Cat #7017782001) for 32 min at room temperature;
7. Anti-HQ HRP (Ventana Medical Systems; Cat #7017936001) for 32 min at room temperature;
8. ChromoMap DAB kit (Ventana Medical Systems; Cat #5266645001) for 8 min at room temperature;
9. Hematoxylin II (Ventana Medical Systems; Cat #5277965001) for 4 min at room temperature.

Immunolabeled slides were dehydrated in graded alcohols, cleared in xylene, and cover-slipped prior to imaging with a Leica AT2 slide scanner (Leica Biosystems, Buffalo Grove, IL, USA). 

Total MMP9 IHC was conducted with Abcam rabbit monoclonal AB76003.

Human sera (not matched) were collected by Bioreclamation Inc (Long Island, NY, USA). Pro-MMP9 levels in human sera were determined with the human MMP9 Quantikine kit (R&D Systems; Cat #DMP900), using a standard four-parameter logistic fit.

## 3. Results

### 3.1. MMP9 Cleaved at Phe107 Is Proteolytically Active

**Figure 1:**
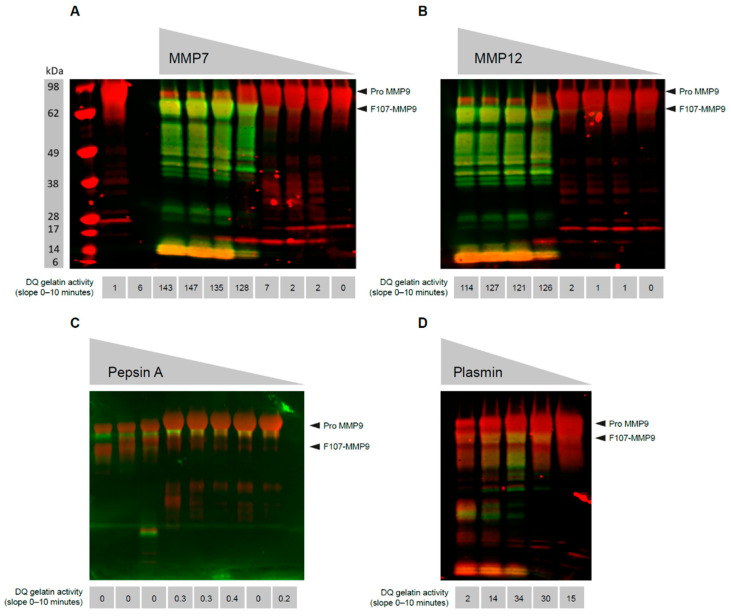
Activation of MMP9 by distinct proteases in vitro. Western blots for pro-MMP9 (92 kDa, red) and active MMP9 (84 kDa, green) after incubation with active (**A**) MMP7, (**B**) MMP12, (**C**) pepsin A, and (**D**) plasmin. Below each lane is the MMP9 activity towards gelatin expressed as gelatin fluorescence (arbitrary units) per minute. DQ, dye-quenched; MMP, matrix metalloproteinase.

Proteolysis is necessary to remove the pro-domain of MMP9, but not all proteolysis steps are sufficient to render an active form of the protein [22]. As multiple proteases are capable of proteolyzing MMP9 [31,32,33,34], we attempt to determine the proteolysis events that are activating. MMP3 [32], MMP7, and MMP12 treatment of pro-MMP9 produce a limited mixture of MMP9 proteolysis products, in which the dominant component is an MMP9 species cleaved at the pro:catalytic domain junction site, Arg106:Phe107 (Figure 1A,B). Pepsin A digestion also results in a limited set of proteolysis events that result in a peptide mix containing MMP9 cleaved at Phe107 (Figure 1C). Plasmin treatment results in a diverse array of MMP9 cleavage products, only some of which contain F107-MMP9 (Figure 1D). Although analysis of these digestions by Western blot can identify F107-MMP9, functional analysis of the same reactions is necessary to assess the species that are active. Using gelatin proteolysis reactions, proteolysis of dye-quenched gelatin by MMP9 occurs in the reactions in which F107-MMP9 is observed by Western blot, whereas MMP9 that forms proteolyzed at sites other than Phe107 is not active. As a control, reactions run in parallel with the maximum concentration of activating protease but lacking MMP9 confirm that the gelatinase activity is MMP9 specific. Therefore, proteolysis outside the pro:catalytic domain junction Arg106:Phe107 is not sufficient to render an active MMP9.

### 3.2. Generation of a mAb That Recognizes the N-Terminus of Active MMP9

**Figure 2:**
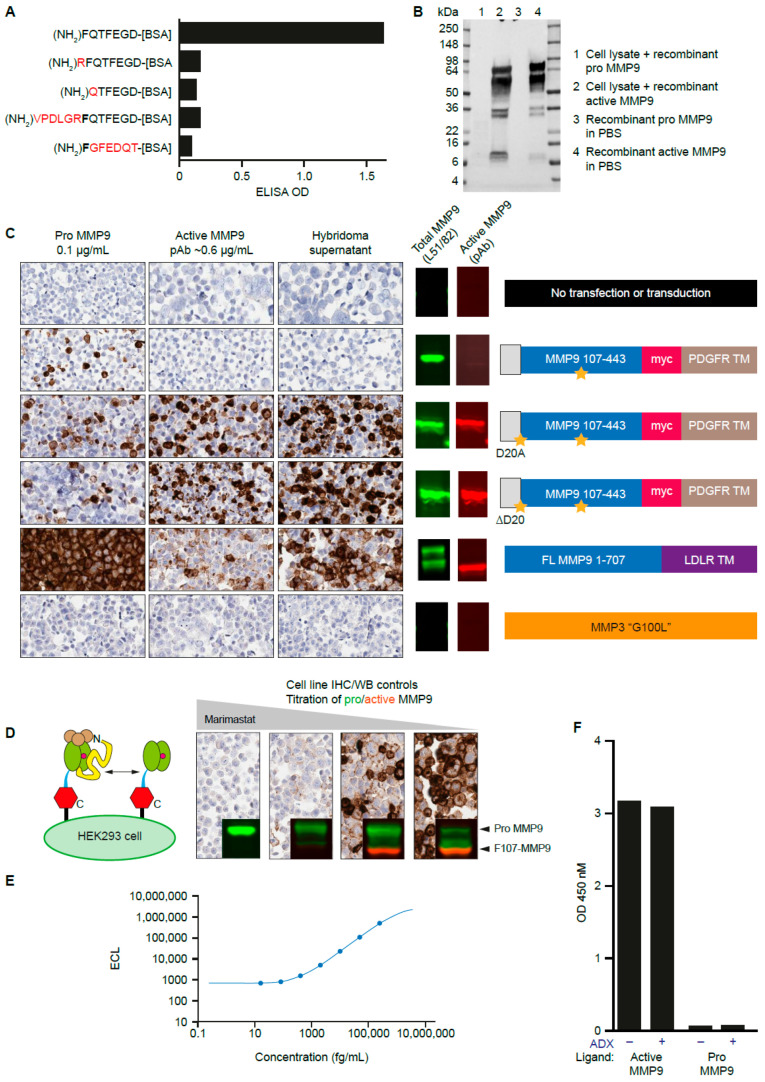
Discovery and characterization of antibodies directed at the active MMP9 neoepitope. (**A**) Hybridoma supernatants specific for the N-terminus of MMP9 after cleavage between Arg106 and Phe107 were identified by direct ELISA toward the corresponding on-target and off-target peptides. (**B**) Pro-MMP9 and active-MMP9 protein standards in buffer or cell lysate were probed with candidate hybridoma supernatants by Western blot. The multiple bands represent the multiple degradation products of MMP9. (**C**) Antibodies that reacted with FFPE cell pellets engineered to contain the active MMP9 neoepitope were identified. Orange stars indicate point mutations, either at the signal peptide junction with F107 (D20A or D20 deletion engineered to generate the F107 neoepitope; see Appendix A) or a mutation in the catalytic domain rendering MMP9 inactive. PDGFR TM and LDLR TM refer to transmembrane domains of PDGFR and LDLR, respectively. MMP3 G100L is an active form of MMP3, a negative control. Molecular weights of bands shown correspond to Figure 1. (**D**) Specificity of the candidate IHC antibodies were determined using cell pellets in which the pro-MMP9:active MMP9 ratio was titrated using marimastat (0.025–2.5 μM). Inset Western blot images fall between the 98 and 62 kDa molecular weight markers. (**E**) A sandwich ELISA using anti–active MMP9 antibodies paired with total MMP9 antibody detected active MMP9 in diluent with a sensitivity <1 pg/mL. (**F**) ADX does not interfere with the sandwich ELISA for active MMP9. ADX, andecaliximab; BSA, bovine serum albumin; ECL, electrochemiluminescence; ELISA, enzyme-linked immunosorbent assay; FFPE, formalin-fixed/paraffin-embedded; IHC, immunohistochemistry; LDLR, low-density lipoprotein receptor; MMP, matrix metalloproteinase; OD, optical density; PDGFR, platelet-derived growth factor receptor; PBS, phosphate-buffered saline; TM, transmembrane; WB, Western blot.

To generate an antibody specific for the mature form of MMP9, rabbits were immunized with the peptide (NH_2_)-FQTFEGDC as previously described [35]. This unique sequence, which is not found in other proteases, represents the neoepitope (the non-native amino terminus of the catalytic domain after the pro-domain is proteolyzed during activation) exposed due to MMP9 cleavage between Arg106 and Phe107 [20]. In total, 40,000 hybridoma supernatants were screened for binding to the immunizing peptide, and positive clones were counterscreened against related peptides (Figure 2A). HEK293 lysates spiked with recombinant pro-MMP9 or MMP3-digested MMP9 (active MMP9) were analyzed by Western blot, with the top hybridoma supernatants used in place of a primary antibody, and were found to react specifically with active MMP9 (Figure 2B). Hybridomas producing antibodies specific for the MMP3-digested MMP9 were identified and selected for subcloning and subsequent antibody purification. 

To further characterize these antibodies, cell lines were generated that expressed pro-MMP9 or active MMP9. This was achieved by transfecting an MMP9 construct in which the signal peptidase site was engineered to produce mature MMP9 starting at Phe107 in HEK293 cells (Figure 2C). The parental transgene contained was engineered to produce an MMP9 mutant with an N-terminal aspartate as a negative control. Two mutants, D20A and ∆D20, successfully produced F107-MMP9 with the intended N-terminal phenylalanine. Additionally, HT1080 cells were transfected with full-length MMP9 with a c-terminal transmembrane domain. The transmembrane domain brings MMP9 in proximity to other transmembrane proteases, such as MMP14 [36], resulting in activation. This activation can be blocked with a broad-spectrum small-molecule MMP inhibitor (marimastat) [37] or ADX [27,28]. These cells were pelleted, fixed in formalin, and assembled into a microarray for assessment of hybridoma supernatants under IHC conditions (Figure 2D). Antibodies specific for the cell lines producing active, but not pro, MMP9 were identified and further evaluated as candidate IHC antibodies. 

The hybridomas producing antibodies specific for active MMP9 were sequenced, and recombinant mAbs were expressed in HEK293 cells. The ELISA performance of the resulting antibody, AB006988, was also assessed for binding to recombinant active MMP9 (Figure 2E,F). Further, ADX, an inhibitory antibody that binds to the active domain of MMP9 [27,28], did not block binding of AB006988 to active MMP9. AB006988 was found to selectively detect active MMP9, but not the inactive MMP9 pro-form, by Western blot, ELISA, and IHC. 

### 3.3. Active MMP9 Is Detectable in IBD

Pro- and active MMP9 are observed in homogenates from diseased, but not healthy, colon tissues.

**Figure 3:**
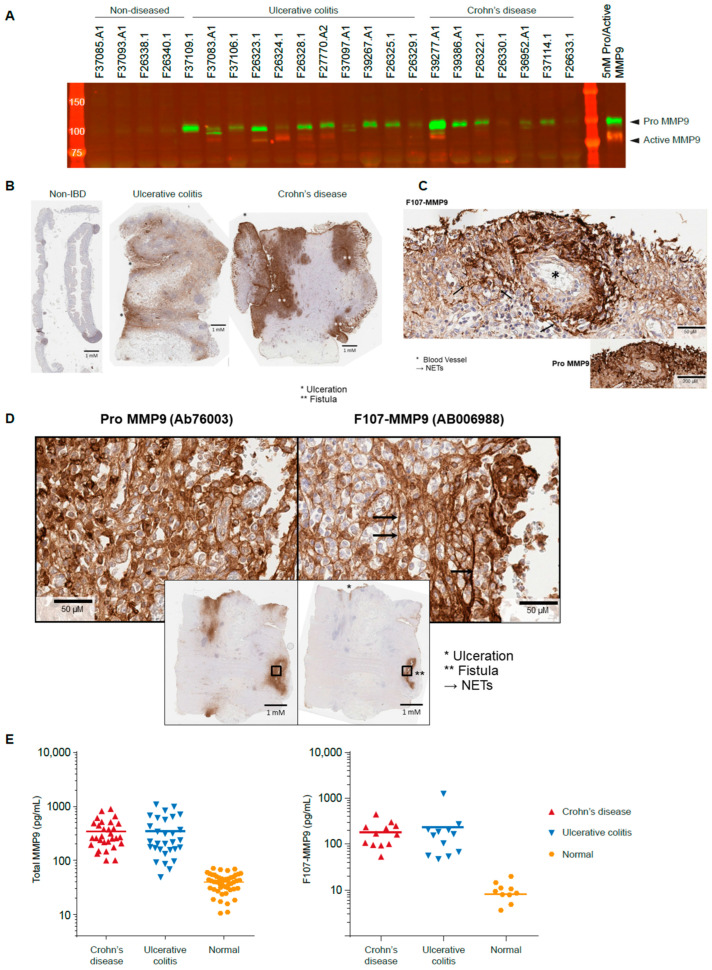
Pro- and active MMP9 in human IBD specimens. (**A**) Homogenized colon tissue from patients with ulcerative colitis and Crohn’s disease contain distinct levels of pro-MMP9 and active MMP9. (**B**) Pro-MMP9 is widespread in diseased colon tissue. (**C**) F107-MMP9 is focal and associated with diseased blood vessels and (**D**) fistulae. (**E**) Serum from patients with ulcerative colitis and Crohn’s disease contains higher levels of pro-MMP9 and active MMP9 than serum from healthy control donors. IBD, inflammatory bowel disease; MMP, matrix metalloproteinase; NETs, neutrophil extracellular traps.

As total MMP9 (both active and pro-MMP9) is shown to be highly expressed in the context of IBD [27], we next investigated the location of active MMP9 expression using the AB006988 antibody. Homogenized samples from diseased or non-diseased regions of whole-colectomy specimens from IBD patients were analyzed. Active MMP9 is not observed in the non-diseased colon specimens. In contrast, the F107-MMP9 species is observed in certain diseased specimens taken from patients with ulcerative colitis and Crohn’s disease (Figure 3A). The active MMP9 band is observed at the expected molecular weight. Levels of active MMP9 do not appear to correlate with levels of parental MMP9, as specimens with the most intense total MMP9 staining are not necessarily the specimens with the most intensely active MMP9 band.

### 3.4. Active MMP9 Expression Is Pronounced in Inflamed Blood Vessels and Fistulae

IHC reveals that pro-MMP9 is mainly confined to cells resembling resident tissue macrophages in healthy tissues, while its presence is widespread in diseased tissues (Figure 3B). In contrast, active MMP9 is not detected in the healthy gut, and its presence in diseased tissue is focal and not widespread. Intense total MMP9 immunostaining is observed around inflamed blood vessels and sites of ulceration of the epithelial barrier (Figure 3C). Intense staining of active MMP9 is observed around fistulae (Figure 3D). Pro-MMP9 in diseased colon tissue appears to be both intracellular and extracellular, consistent with the production and secretion of the pro-MMP9 isoform. Active MMP9, in contrast, appears predominantly extracellularly, with little evidence of intracellular or granular staining. This suggests that MMP9 is activated after or during secretion, where it likely comes into contact with the extracellular or membrane-bound proteases reported to activate it. Active MMP9 is not detected in a number of cases with pronounced pro-MMP9 staining, indicating that either a set fraction of pro-MMP9 is not predictably activated, or the activated isoform is below thresholds of detection in this setting. 

### 3.5. Active MMP9 Is Observed in IBD Sera

We next assessed circulating levels of active and pro-MMP9 in the sera of patients with IBD. Using recombinant protein calibrators, this assay is able to report absolute concentration of each MMP9 isoform in human serum. Both isoforms of MMP9 are elevated in IBD sera as compared with normal sera (Figure 3E). The amount of active MMP9 is consistently 1000-fold lower than the concentration of pro-MMP9. Thus, active MMP9 levels cannot be predicted from pro-MMP9 quantification.

### 3.6. Active MMP9 Expression in HS

**Figure 4:**
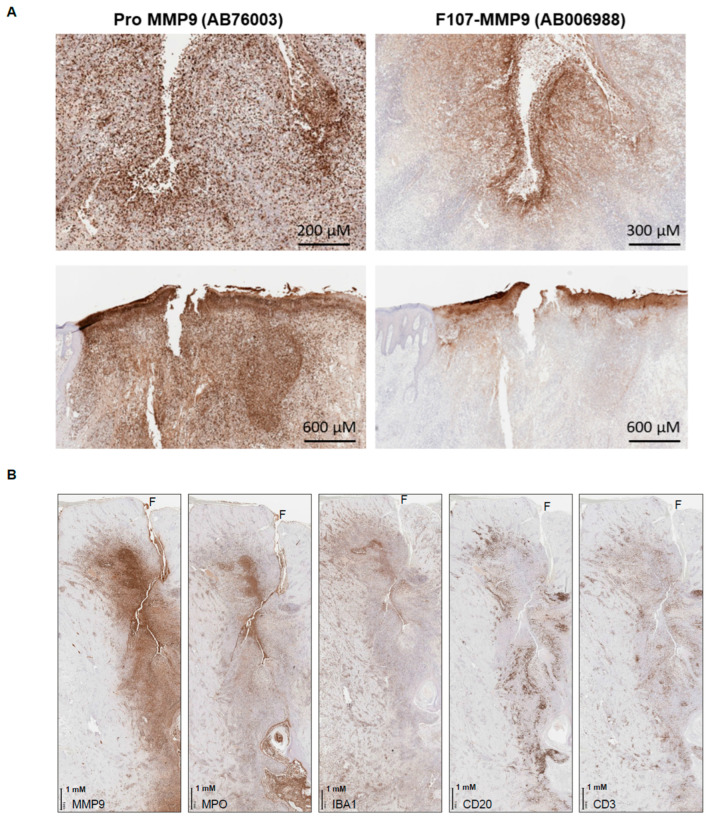
Pro- and active MMP9 in hidradenitis skin specimens. (**A**) Pro-MMP9 is widespread in the skin of patients with hidradenitis, while active MMP9 is more focal and associated with ruptured epidermis (top) and ruptured hair follicles (bottom). (**B**) Active MMP9 and myeloid cells (MPO+ neutrophils and IBA1+ macrophages) are found proximal to the rupture in the skin, while lymphocytes (CD20+ B cells and CD3+ T cells) are secondary. F, fistula; IBA1, anti-ionized calcium-binding adaptor protein-1; MMP, matrix metalloproteinase; MPO, myeloperoxidase.

Given the association of active MMP9 with fistulae in IBD tissue, we hypothesized that other diseases involving non-traumatic tears in tissue might also have high active-MMP9 levels. HS is an example of such a disease, in which fissures of the dermis are reported to emerge between ruptured hair follicles [38]. Assessment of six cases reveals a highly similar pattern of intense, focal, and extracellular active-MMP9 expression around the fissures (Figure 4A). Expression of pro-MMP9 is again more widespread and includes both intracellular and extracellular staining patterns. The active MMP9 expression is associated with the presence of neutrophils and macrophages, as evidenced by myeloperoxidase and anti-ionized calcium-binding adaptor protein-1 staining, respectively. B cells and T cells—as evidenced by CD20 and CD3 staining, respectively—are peripheral to the lesions (Figure 4B). Production of active and TIMP-free MMP9 by neutrophils has been previously reported [1], and is consistent with our IHC observations.

## 4. Conclusions

Although tools and assays to detect the pro-forms of proteases such as MMP9 exist [15], reagents to enable deeper insight into protease activation and activity in disease settings have been limited. This report describes specific techniques capable of reliably measuring an active form of MMP9 in multiple types of biospecimens. Generating the active forms of proteases through cellular processing or via recombinant manipulation has also been described previously [20]. Nonetheless, within the body of results generated with these methods, there are instances of confusion and inconsistency in the identification of different protease species. For example, MMP9 proteolyzed to an ~82 kDa species may or may not correspond with the active-MMP9 form, as detailed in Figure 1. Specific and well-characterized reagents and assays can aid in experimental design and clarify interpretation.

While MMP9 is capable of proteolyzing a relatively broad array of proteins [39,40], its levels in tissue and circulation have often been confounding or difficult to functionally interpret. Reports suggesting that pro-MMP9 can be found at concentrations >1 μg/mL in certain specimens [41,42] have grappled with how to interpret such high abundance. This report clarifies that pro-MMP9 is indeed quite abundant, but detection of an active species is notably more limited, suggesting that its activation is tightly controlled. The focal localization of active-MMP9 IHC staining also suggests a mechanism by which MMP9 can overcome endogenous inhibitors such as TIMP and α2-macroglobulin. Clusters of active MMP9 in dense foci may be in simple stoichiometric excess of its inhibitors at these defined tissue sites. Further studies quantifying MMP9 and TIMP levels could address this mechanism directly.

In this study, only MMP9 cleaved between Arg106 and Phe107 performs as an active protease in the functional assay reported. This represents the predominant cleavage site in vitro, although it is impossible to rule out the existence of some other active-MMP9 form in human tissue with a distinct N-terminus. The pro- and active- MMP9 isoforms are likely to differ in their cellular locations and in their turnover patterns. Pro-MMP9 is stored in intracellular granules at appreciable quantities. Active MMP9, in contrast, is not stored intracellularly [43,44] and is reportedly short-lived [45]. Thus, an issue to keep in mind when carrying out relative quantifications of both protein species is that readings of pro-MMP9 levels may integrate total amounts accumulated over a longer time frame than any assessment of the active form. 

The ultimate physiological consequences of MMP9 activation are likely dependent on the cellular and biochemical environment. The specific substrates of MMP9 are consequential for its function, and they are likely to vary in distinct disease settings [39,40], and the presence and levels of specific MMP9-activating proteases may also vary. In the HS cases examined in this report, MMP9 expression and activation is observed in areas with neutrophils and macrophages. The local production of MMP9 substrates in that context provides a potential mechanism for a feed-forward loop that promotes inflammation. In other contexts, such as the tumor microenvironment, MMP9 expression is associated with a distinct subset of infiltrating myeloid cells and tumor epithelial cells [3,46,47]. Hence, the local substrates available for MMP9 cleavage are likely distinct and may drive distinct physiology. Thus, the translation, secretion, and activation of MMP9, paired with the local production and presentation of substrates, may work in concert to regulate specific pathological processes.

## Data Availability

Data are contained within the article.

## Appendix A. Transgene Amino Acid Sequences Used in This Report

>MMP3G100L
mkslpillllcvavcsaypldgaargedtsmnlvqkylenyydlkkdvkqfvrrkdsgpvvkkiremqkflglevtgkldsdtlevmrkprcLvpdvghfrtfpgipkwrkthltyrivnytpdlpkdavdsavekalkvweevtpltfsrlyegeadimisfavrehgdfypfdgpgnvlahayapgpgingdahfdddeqwtkdttgtnlflvaaheighslglfhsantealmyplyhsltdltrfrlsqddingiqslygpppdspetplvptepvppepgtpancdpalsfdavstlrgeilifkdrhfwrkslrklepelhlissfwpslpsgvdaayevtskdlvfifkgnqfwairgnevragyprgihtlgfpptvrkidaaisdkeknktyffvedkywrfdekrnsmepgfpkqiaedfpgidskidavfeefgffyfftgssqlefdpnakkvthtlksnswlnc

>MMP9 TM
mslwqplvlvllvlgccfaaprqrqstlvlfpgdlrtnltdrqlaeeylyrygytrvaemrgeskslgpallllqkqlslpetgeldsatlkamrtprcgvpdlgrfqtfegdlkwhhhnitywiqnysedlpraviddafarafalwsavtpltftrvysrdadiviqfgvaehgdgypfdgkdgllahafppgpgiqgdahfdddelwslgkgvvvptrfgnadgaachfpfifegrsysacttdgrsdglpwcsttanydtddrfgfcpserlytrdgnadgkpcqfpfifqgqsysacttdgrsdgyrwcattanydrdklfgfcptradstvmggnsagelcvfpftflgkeystctsegrgdgrlwcattsnfdsdkkwgfcpdqgyslflvaahefghalgldhssvpealmypmyrftegpplhkddvngirhlygprpepeprppttttpqptapptvcptgpptvhpserptagptgppsagptgpptagpstattvplspvddacnvnifdaiaeignqlylfkdgkywrfsegrgsrpqgpfliadkwpalprkldsvfeeplskklfffsgrqvwvytgasvlgprrldklglgadvaqvtgalrsgrgkmllfsgrrlwrfdvkaqmvdprsasevdrmfpgvpldthdvfqyrekayfcqdrfywrvssrselnqvdqvgyvtydilqcpedGTALSIVLPIVLLVFLCLGVFLLWKNWRLKN

>>MMP9_dead_PDGFR
METDTLLLWVLLLWVPGSTGDfqtfegdlkwhhhnitywiqnysedlpraviddafarafalwsavtpltftrvysrdadiviqfgvaehgdgypfdgkdgllahafppgpgiqgdahfdddelwslgkgvvvptrfgnadgaachfpfifegrsysacttdgrsdglpwcsttanydtddrfgfcpserlytrdgnadgkpcqfpfifqgqsysacttdgrsdgyrwcattanydrdklfgfcptradstvmggnsagelcvfpftflgkeystctsegrgdgrlwcattsnfdsdkkwgfcpdqgyslflvaahAfghalgldhssvpealmypmyrftegpplhkddvngirhlygprpepeEQKLISEEDLNAVGQDTQEVIVVPHSLPFKVVVISAILALVVLTIISLIILIMLWQKKPR

>>MMP9_dead_PDGFR_D20A
METDTLLLWVLLLWVPGSTGAfqtfegdlkwhhhnitywiqnysedlpraviddafarafalwsavtpltftrvysrdadiviqfgvaehgdgypfdgkdgllahafppgpgiqgdahfdddelwslgkgvvvptrfgnadgaachfpfifegrsysacttdgrsdglpwcsttanydtddrfgfcpserlytrdgnadgkpcqfpfifqgqsysacttdgrsdgyrwcattanydrdklfgfcptradstvmggnsagelcvfpftflgkeystctsegrgdgrlwcattsnfdsdkkwgfcpdqgyslflvaahAfghalgldhssvpealmypmyrftegpplhkddvngirhlygprpepeEQKLISEEDLNAVGQDTQEVIVVPHSLPFKVVVISAILALVVLTIISLIILIMLWQKKPR

>>MMP9_dead_PDGFR_ΔD20
METDTLLLWVLLLWVPGSTGfqtfegdlkwhhhnitywiqnysedlpraviddafarafalwsavtpltftrvysrdadiviqfgvaehgdgypfdgkdgllahafppgpgiqgdahfdddelwslgkgvvvptrfgnadgaachfpfifegrsysacttdgrsdglpwcsttanydtddrfgfcpserlytrdgnadgkpcqfpfifqgqsysacttdgrsdgyrwcattanydrdklfgfcptradstvmggnsagelcvfpftflgkeystctsegrgdgrlwcattsnfdsdkkwgfcpdqgyslflvaahAfghalgldhssvpealmypmyrftegpplhkddvngirhlygprpepeEQKLISEEDLNAVGQDTQEVIVVPHSLPFKVVVISAILALVVLTIISLIILIMLWQKKPR
